# Flexible oxidation of styrene using TBHP over zirconia supported mono-copper substituted phosphotungstate

**DOI:** 10.1039/c9ra04892h

**Published:** 2019-09-03

**Authors:** Rajesh Sadasivan, Anjali Patel

**Affiliations:** Polyoxometalates & Catalysis Laboratory, Department of Chemistry, Faculty of Science, The Maharaja Sayajirao University of Baroda Vadooara Gujarat India anjali.patel-chem@msubaroda.ac.in

## Abstract

A heterogeneous catalyst comprising mono-copper substituted phosphotungstate and hydrous zirconia was synthesized using wet impregnation method, characterized by various physico-chemical techniques and evaluated for solvent-free oxidation of styrene using TBHP as oxidant. Various reaction parameters like time, catalyst amount, amount of TBHP and temperature were optimized with focus on optimum selectivity of styrene-oxide. Further, the catalytic activity was compared with that of unfunctionalized PW_11_Cu to understand the role of the support. Finally, the role of each component of the reaction was clearly elucidated by a detailed kinetic study of the reaction using both the catalysts.

## Abstract 2

Mono-copper substituted phosphotungstate was supported on hydrous zirconia by wet impregnation, characterized and evaluated for its catalytic activity in the solvent-free flexible oxidation of styrene using TBHP as oxidant.

## Graphical abstract

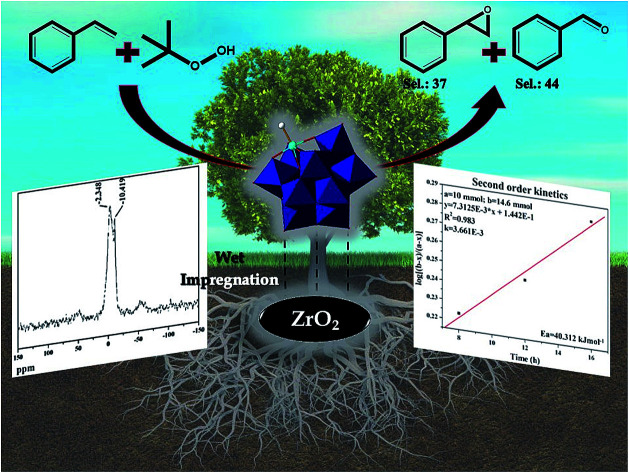

## Introduction

The catalytic oxidation of styrene has gained much attention in industry as well as in academia, as it forms the basis for producing various intermediates, bulk and fine chemicals. The humongous use of benzaldehyde in the flavouring, perfumery, pharmaceutical and dye industries makes it one of the most valuable aromatic aldehydes.^1^ Moreover, styrene oxide produces styrene glycol and its derivatives which are used in surface coatings and cosmetics.^2^ It is therefore, not surprising that to date, various homogeneous and heterogeneous catalysts such as metal complexes,^3–6^ ionic liquids,^7,8^ metal–organic frameworks (MOFs),^9–11^ zeolites,^12,13^ metal nanoparticles (NPs),^14,15^ metal oxides,^16–18^*etc.* have been used for the oxidation of styrene.^1^

The greatest disadvantage of catalysts in bulk form is their low surface area, which reduces the efficiency of the catalyst.^19^ Moreover, homogeneous catalysts always have the drawback of limited reusability as they cannot be easily be recycled. In order to overcome these drawbacks, anchoring of catalytic materials on various supports *via* dative, covalent, acid–basic, or electrostatic interactions has emerged as an upcoming research area.^20–23^ Amongst all materials, polyoxometalates (POMs) anchored to various supports have received increasing attention as they can undergo fast reversible multi-electron redox transformations under mild conditions. Use of different supports like mesoporous silica, zirconia, zeolites, and alumina to immobilize POMs have been explored for oxidation of styrene.

In 2008, Tangestaninejad *et al.* synthesized di-vanadium substituted phosphomolybdate supported on mesoporous MCM-41 and used the same for oxidation of various cyclic and aromatic alkenes to their respective aldehydes and ketones under UV radiations.^24^ In the same year, Guo *et al.* synthesized periodic mesoporous composite catalysts, [(*n*-C_4_H_9_)_4_N]_4_[γ-SiW_10_O_34_(H_2_O)_2_]/SBA-15], and used for epoxidation of styrene.^25^ In 2009, various transition metal substituted polyoxotungstates, K_10−*n*_X^*n*+^MW_11_O_39_ (X = P/Si, M = Co/Ni/Cu/Mn), were supported on Schiff base modified SBA-15 by Guo *et al.* These were further used for selective oxidation of styrene to benzaldehyde.^26^ Later, in 2010, Kholdeeeva *et al.* synthesized MIL-101 supported phosphotungstates [(PW_4_O_24_)^3−^ and (PW_12_O_40_)^3−^] and used them as sustainable and reusable catalysts for selective epoxidation of styrene.^27^ In 2013, Balula *et al.* reported the synthesis of SiW_11_Co and SiW_11_Fe immobilized onto an amine functionalized SBA-15 and investigated their catalytic property for oxidation of styrene and geraniol.^28^ In the following year, the same group reported the synthesis of a zinc substituted polyoxometalate, [PW_11_Zn-(H_2_O)O_39_]^5−^ (PW_11_Zn), and encapsulation into silica nanoparticles using a cross-linked organic–inorganic core. They further used the same as a versatile catalyst for the oxidation of styrene.^29^ Selective oxidation of styrene to epoxystyrene was carried out by Zhang *et al.* in the same year using phosphomolybdic acid supported on 1-triethoxysilylpropyl-3-methylimidazoliumchloride IL modified MCM-41.^30^ Our group has worked extensively in this field wherein, a wide range of POMs have been anchored on various metal-oxide as well as silica based supports and used for styrene oxidation. Parent as well as mono-lacunary phosphotungstates, -molybdates and silicotungstates anchored to zirconia, alumina, mesoporous silica and zeolites were used for oxidation of styrene with green oxidants like hydrogen peroxide and molecular oxygen.^31–37^

A literature survey showed that there are no reports available where copper substituted phosphotungstate is supported on zirconia. Further, all the above mentioned reports have used either H_2_O_2_ or molecular O_2_ as oxidants. No accounts are available where *tert*-butyl hydroperoxide (TBHP) has been used as the oxidant for oxidation of styrene, despite it being an equally green and environmentally benign oxidant.

In the present work, we have anchored mono-copper substituted POM to hydrous zirconia by wet impregnation technique and carried out characterization of the supported catalyst by various physico-chemical techniques. We report a comparative study of the catalytic activity of both, the supported as well as unsupported catalysts for the oxidation of styrene using TBHP as the oxidant and discuss the role of the support. Leaching and heterogeneity tests as well as recycle studies have been carried out. Finally, the kinetics of the reaction using both catalysts has also been studied in detail.

## Experimental

### Materials

All chemicals used were of A. R. grade. 12-tungstophosphoric acid, copper chloride dihydrate, cesium chloride, styrene, dichloromethane, 70% *tert*-butyl hydroperoxide, liq. NH_3_ and sodium hydroxide were obtained from Merck. Zirconium oxychloride was procured from Loba Chemie. All chemicals were used as received.

### Synthesis of zirconia

Hydrous zirconia was synthesized using a technique previously reported by our group.^38^ To an aqueous solution of ZrOCl_2_·8H_2_O, ammonia solution was added drop-wise upto pH 8.5. This was aged at 100 °C for 1 h in a water bath, filtered, washed and dried at 100 °C for 10 h. The obtained material was designated ZrO_2_.

### Synthesis of mono-copper substituted phosphotungstate^39^

H_3_PW_12_O_40_·*n*H_2_O (2.88 g; 1 mmol) was dissolved in 10 mL of water and the pH of the solution was adjusted to 4.8 using supersaturated NaOH solution. The solution was heated to 90 °C with stirring. CuCl_2_·2H_2_O (0.17 g; 1 mmol) was dissolved in minimum amount of water and then added drop-wise to the hot POM solution. This was then air-refluxed for 1.5 h at 90 °C, filtered hot, and solid CsCl (0.5 g) was immediately added. The resulting greenish blue precipitates were filtered, dried at room temperature and designated as PW_11_Cu.

### Synthesis of mono-copper substituted phosphotungstate supported on zirconia

30% PW_11_Cu supported over zirconia was synthesized by wet impregnation method. To the aqueous solution of PW_11_Cu (0.3 g/30 mL), 1 g ZrO_2_ was added and dried at 100 °C for 10 h and the resulting material was designated 30% PW_11_Cu/ZrO_2_. On similar lines, 10%, 20% and 40% PW_11_Cu/ZrO_2_ were prepared taking 0.1 g/10 mL, 0.2 g/20 mL and 0.4 g/40 mL aqueous solutions of PW_11_Cu respectively and designated as 10% PW_11_Cu/ZrO_2_, 20% PW_11_Cu/ZrO_2_ and 40% PW_11_Cu/ZrO_2_ respectively.

### Acidity determination by potentiometry

The different types of acidic sites were determined by potentiometric titrations using *n*-butyl amine.^40^ 0.50 g of the synthesized material was suspended in 50 mL acetonitrile and aged at 25 °C. To this, 0.1 mL of 0.05 N *n*-butyl amine in acetonitrile was added at regular time intervals and the potential (mV) after each addition was recorded.

### Characterization

The synthesized material was characterized for its acidic strength, and also by thermo gravimetric-differential thermal analysis (TG-DTA), BET surface area analysis, Temperature Programmed Reduction (TPR), FT-IR spectroscopy, FT-Raman spectroscopy, ^31^P MAS NMR Spectroscopy, Powder XRD and ESR Spectroscopy. Adsorption–desorption analysis for specific surface area calculations was carried out in the Micrometrics ASAP 2010 instrument at −196 °C. TGA was carried out using Mettler Toledo Star SW 7.01 instrument up to 550 °C. The TPR studies were investigated in a self-made reactor set-up with a quartz reactor vessel. 50 mg of sample was taken and heated up to 800 °C and the linear ramping rate was 10 °C min^−1^ with 5% (35 mL min^−1^) H_2_/Ar flow for 60 min. the consumption of H_2_ gas was monitored using GC instrument equipped with TCD (m/s, CIC Instruments, India). FT-IR spectra of the sample were obtained by using the KBr pellet on the Perkin Elmer instrument. ^31^P MAS NMR was recorded in JOEL ECX 400 MHz High Resolution Multinuclear FT-NMR spectrometer for solids. ESR spectra were recorded on a Varian E-line Century series X-band ESR spectrometer (liquid nitrogen temperature and scanned from 2000 to 3000 Gauss).

### Catalytic reaction

Oxidation of styrene was carried out using both, unsupported PW_11_Cu as well as PW_11_Cu/ZrO_2_ as catalyst, in a 50 mL batch reactor attached to a double-walled air condenser on a magnetic stirrer and heating plate. Styrene (10 mmol), catalyst and TBHP were added to the substrate. Due to absence of any aqueous media, the catalyst remained heterogeneous. Dichloromethane was used to extract the products after the reaction, which were then analysed using Shimatzu-2014 Gas Chromatograph, using an RTX-5 capillary column. The products were identified by comparison with authentic samples.

### Leaching test

Polyoxometalates can be easily characterized by a clear heteropoly blue colour when reacted with a mild reducing agent like ascorbic acid. This method was used to check for leaching of PW_11_Cu from the support.

## Results and discussion

### Catalyst characterization

Absence of blue colour on reaction with ascorbic acid denoted that leaching of PW_11_Cu from ZrO_2_ into the reaction medium does not take place, thereby indicating that there are strong interactions between the POM and support.

**Fig. 1:**
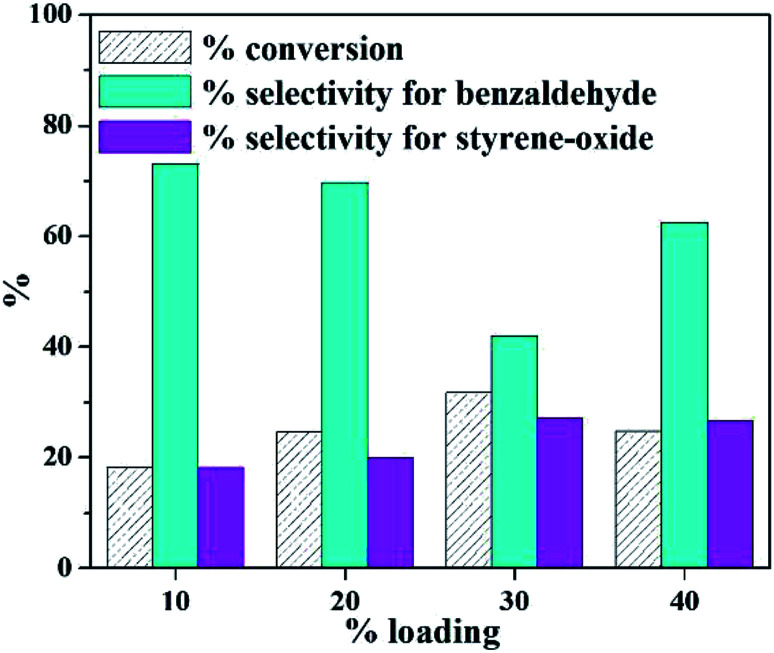
Effect of % loading (catalyst amount – 25 mg; time – 8 h; TBHP – 2 mL; temperature – 60 °C).

In order to find out the optimum amount of loading, a preliminary reaction was carried out at varied % loading of active species (10–40%) and the results are presented in Fig. 1. It is seen that with increase in loading amount up to 30%, there is a steady increase in conversion, as expected. But it is interesting to note a decrease in selectivity of benzaldehyde with a steady increase in selectivity of styrene-oxide. Beyond 30% loading, there is a decrease in % conversion of styrene, which may be attributed to blocking of catalytic sites due to excess loading.

It is well known that the completion of the reaction to give benzaldehyde depends on the acidity of the catalyst. In the present case, the acidity of the catalyst decreases with increase in % loading of active species. As a result, the selectivity of the intermediate, *i.e.*, styrene-oxide tends to increase. Keeping in mind the importance of epoxide in the chemical industry, 30% PW_11_Cu/ZrO_2_ was optimized and further characterizations as well as optimizations have been carried out using 30% PW_11_Cu/ZrO_2_ designated as only PW_11_Cu/ZrO_2_.

**Fig. 2:**
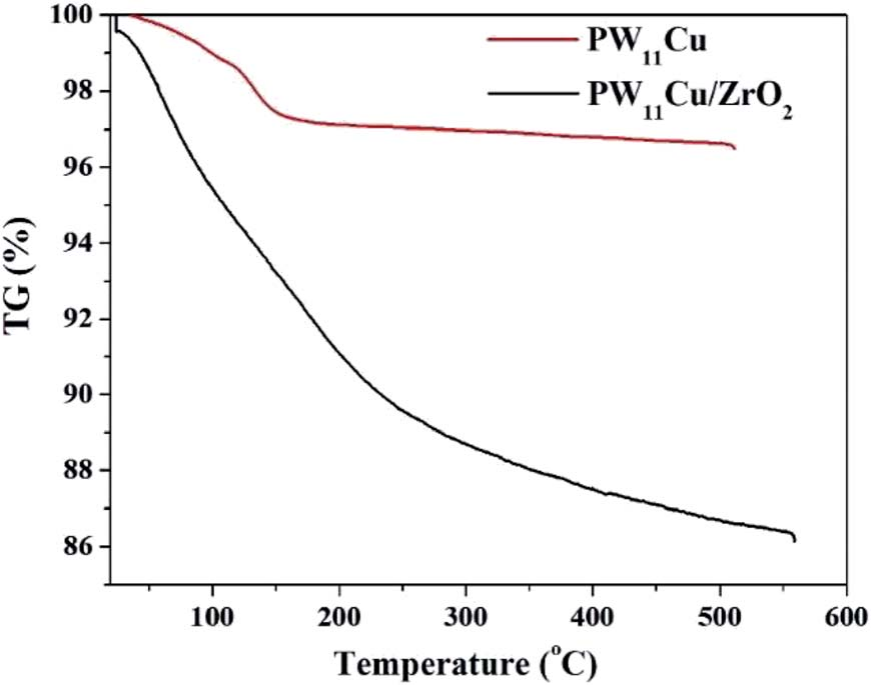
TGA of PW_11_Cu and PW_11_Cu/ZrO2.

TGA of PW_11_Cu/ZrO_2_ (Fig. 2) shows initial weight loss of 4.4% up to 150 °C, which is attributed to adsorbed water. Further weight loss of 7.2% is noticed between 180–350 °C because of water of crystallization. No other substantial weight loss indicates that the synthesized material is stable up to 550 °C.

**Fig. 3:**
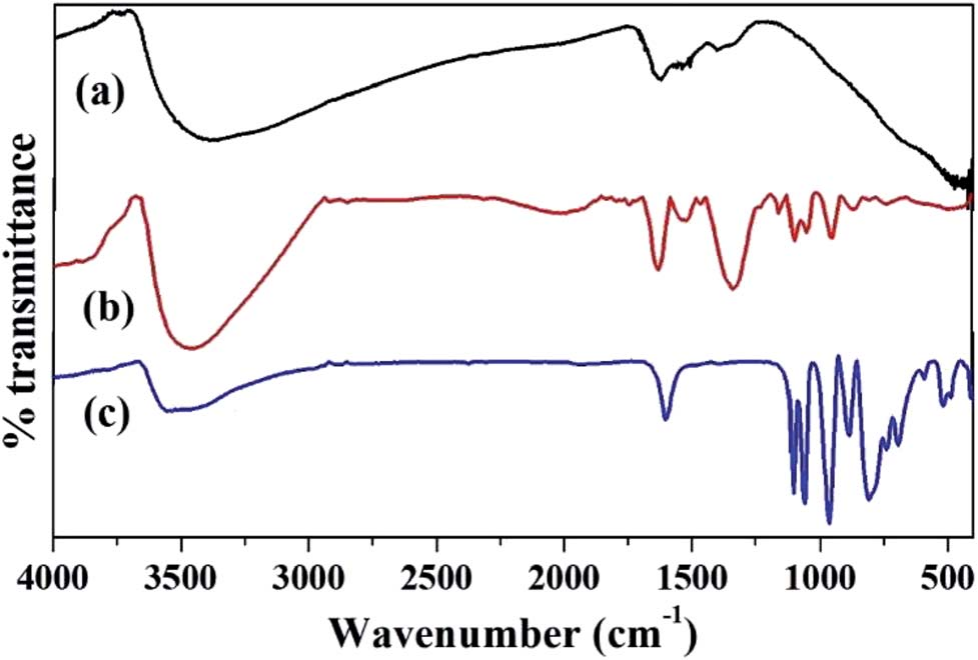
FT-IR spectra of (a) ZrO_2_, (b) PW_11_Cu/ZrO_2_ and (c) PW_11_Cu.

The FT-IR spectra of ZrO_2_, PW_11_Cu and PW_11_Cu/ZrO_2_ are shown in Fig. 3. ZrO_2_ shows bands at 3365, 1625, 1396 and 608 cm^−1^ which are characteristic of asymmetric O–H stretching, H–O–H and O–H–O bending and Zr–O–H bending vibrations respectively.

PW_11_Cu exhibits bands at 1103 and 1060 cm^−1^ corresponding to P–O stretching, 964 cm^−1^ corresponding to W=O stretching, 887 and 810 cm^−1^ corresponding to W–O–W stretching and 516 cm^−1^ corresponding to Cu–O stretching vibrations respectively. The FT-IR spectrum of PW_11_Cu/ZrO_2_ shows characteristic bands of both PW_11_Cu and ZrO_2_. Bands at 1103 and 1064 cm^−1^, 952 cm^−1^ and 813 cm^−1^ corresponding to P–O, W=O and W–O–W stretching vibrations are similar to PW_11_Cu. In addition, broad bands at 3417, 1631 and 1402 cm^−1^ correspond to O–H stretching, H–O–H and O–H–O bending respectively are similar to that of ZrO_2_. The slight shifts observed in frequencies of supported catalyst is attributed to the chemical interaction of PW_11_Cu with the support. However, the Cu–O vibration band is not visible, due to overlapping with the Zr–O–H band and hence confirmed by FT-Raman.

**Fig. 4:**
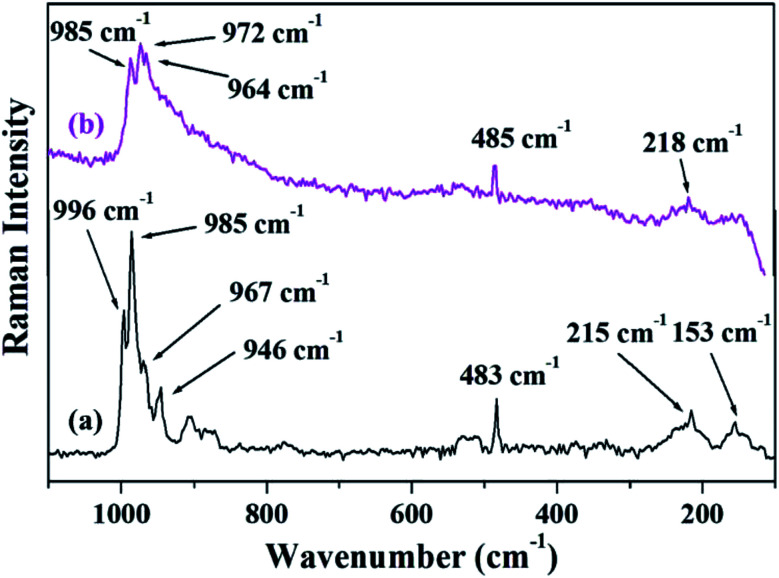
FT–Raman spectra of (a) PW_11_Cu, (b) PW_11_Cu/ZrO_2_.

Fig. 4 shows the FT-Raman spectra of PW_11_Cu as well as PW_11_Cu/ZrO_2_. ZrO_2_ shows a number of broad peaks from 100–800 cm^−1^ associated with long range amorphous state disordering.^41^ The Raman spectra of PW_11_Cu shows peaks at 996 cm^−1^ corresponding to W=O symmetric stretch; 985, 215 and 153 cm^−1^ corresponding to W–O symmetric stretch; and 967 and 946 cm^−1^ corresponding to W–O–W symmetric stretch respectively. Further, an additional peak at 483 cm^−1^ is incorporation of copper in the lacuna of PW_11_. Similarly, Raman spectra of PW_11_Cu/ZrO_2_ shows peaks at 985 cm^−1^ corresponding to W=O symmetric stretch, 972 and 218 cm^−1^ corresponding to W–O symmetric stretch, and 964 cm^−1^ corresponding to W–O–W symmetric stretch respectively. A slight shift as well as decrease in intensity, along with absence of some peaks may be because of the interaction of PW_11_Cu with the support. Thus, FT-IR and FT-Raman confirm that PW_11_Cu remains intact even after impregnation on to the support.

**Fig. 5:**
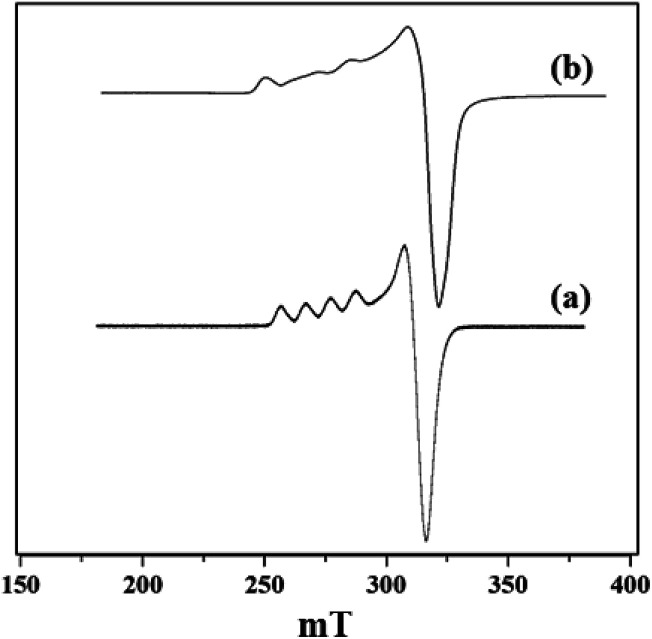
ESR spectra of (a) PW_11_Cu and (b) PW_11_Cu/ZrO_2_.

X-band liquid nitrogen temperature ESR spectra of unsupported PW_11_Cu, ZrO_2_ and PW_11_Cu/ZrO_2_ are presented in Fig. 5. PW_11_Cu gives a four line hyperfine spectrum with *g* = 2.0883 and *g* = 2.4031, typically found in Cu(ii) complexes.^39^ PW_11_Cu/ZrO_2_ retains the hyperfine spectrum of PW_11_Cu, indicating that copper remains in 2+ oxidation state and the environment around Cu(ii) stays intact even after impregnation on to the support. However, the decrease in intensity observed may be due to interaction of PW_11_Cu with the support.

**Fig. 6:**
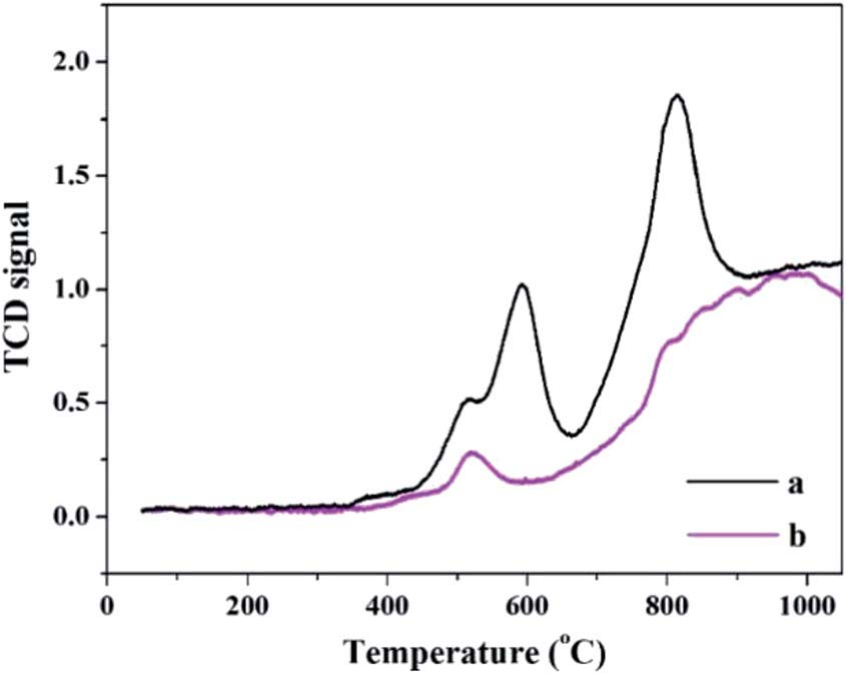
H_2_-TPR spectra of (a) PW_11_Cu and (b) PW_11_Cu/ZrO_2_.

The H_2_-TPR spectra of PW_11_Cu and PW_11_Cu/ZrO_2_ are shown in Fig. 6. Romanelli *et al.* obtained two peaks in the TPR pattern of sodium salt of PW_11_Cu with maxima at 666 °C and 960 °C which have been assigned to reduction of species after anion decomposition.^42^

In the present case, similar maxima are obtained at 592 °C and 819 °C, which is attributed to formation of WO_3_ species.^43^ The decrease in reduction temperature is attributed to increase in the consumption of H_2_ gas due to Cs counter cation.^44^ The lowering of reduction temperature may also be an indication of enhanced oxidation ability.

The H_2_-TPR spectra of PW_11_Cu/ZrO_2_ has a peak with lesser intensity and shows maxima at 518 °C, while the second peak gets overlapped with the reduction peaks of ZrO_2_, which fall in the temperature range of 700–900 °C.^45^ The decrease in intensity as well as reduction temperature may be due to chemical interaction of [PW_11_Cu]^5−^ anions with the support.^42^ This is further confirmed by ^31^P MAS NMR.

**Fig. 7:**
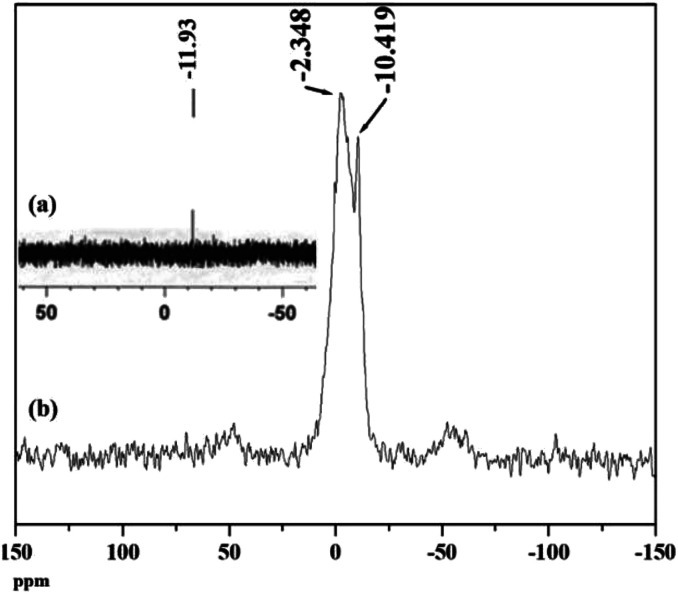
^31^P MAS NMR of (a) PW_11_Cu and (b) PW_11_Cu/ZrO_2_.

^31^P NMR is an important tool to understand the environment around phosphorus in polyoxometalates as well as the interaction of the anion with support.^46^ The ^31^P MAS NMR of PW_11_Cu and PW_11_Cu/ZrO_2_ are presented in Fig. 7. The peak at −10.42 in case of the supported material arises because of the PO_4_ unit of PW_11_Cu. The slight downfield shift may be attributed to the hydrogen bonding between the terminal oxygen of the POM and –OH groups of zirconia. The broad peak at −2.35 is attributed to the formation of [≡Zr–OH_2_]^+^[Cs_4_(PW_11_CuO_39_)]^−^ species.^47–49^ This may be explained as follows. Loading of PW_11_Cu on to ZrO_2_ is carried out by wet impregnation at 100 °C. Dehydration of water molecules of zirconia occurs, which is followed by subsequent entrapment of PW_11_Cu anions within the network of zirconia. Further, due to the acidic medium, ≡Zr–OH gets protonated to form [≡Zr–OH_2_]^+^, which acts as a cation for the anionic (PW_11_CuO_39_)^5−^ species. As a result, a chemical bond formation occurs between the two as opposed to simple physisorption. This, along with hydrogen bonding, ensures that PW_11_Cu does not leach during the catalytic reaction and the catalyst remains intact. Intensity of both the peaks in NMR indicates that while majority of PW_11_Cu form ionic pairs with zirconia, some interact only by hydrogen bonds.

**Fig. 8:**
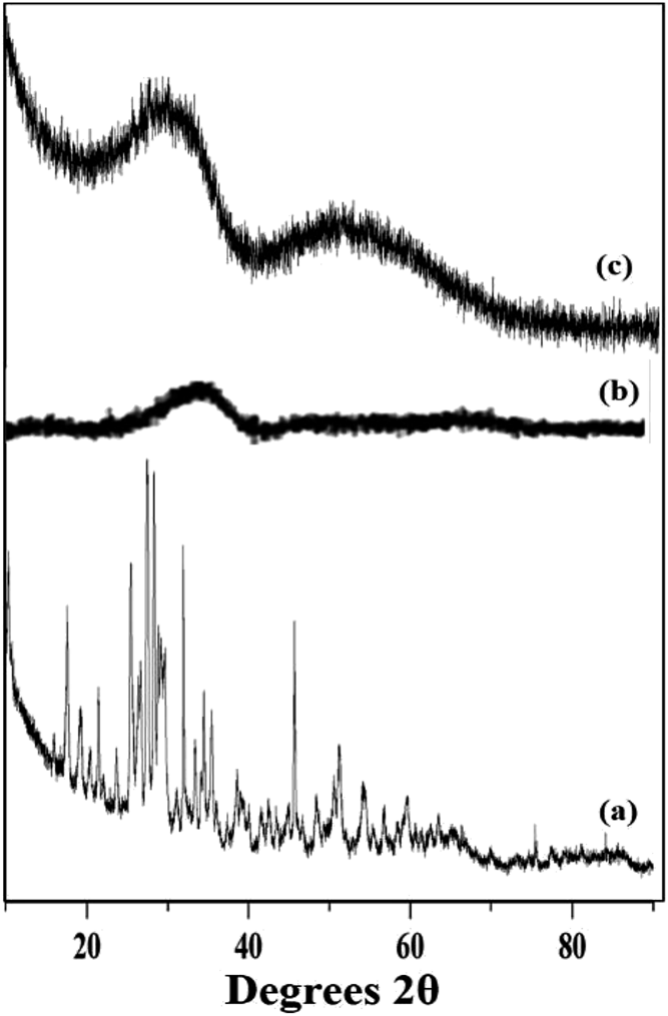
Wide angle powder XRD of (a) PW_11_Cu, (b) ZrO_2_ and (c) PW_11_Cu/ZrO_2_.

The wide angle powder XRD of unsupported PW_11_Cu, ZrO_2_ and PW_11_Cu/ZrO_2_ are presented in Fig. 8. PW_11_Cu shows sharp peaks from 20–30 degrees 2*θ* characteristic to the Keggin structure, with a slight shift due to incorporation of copper. This is further confirmed by sharp peaks at 48 degrees 2*θ* attributed to Cu(ii).^39^ The absence of crystalline peaks in case of PW_11_Cu/ZrO_2_ indicates complete dispersion of PW_11_Cu on to the support.

**Fig. 9:**
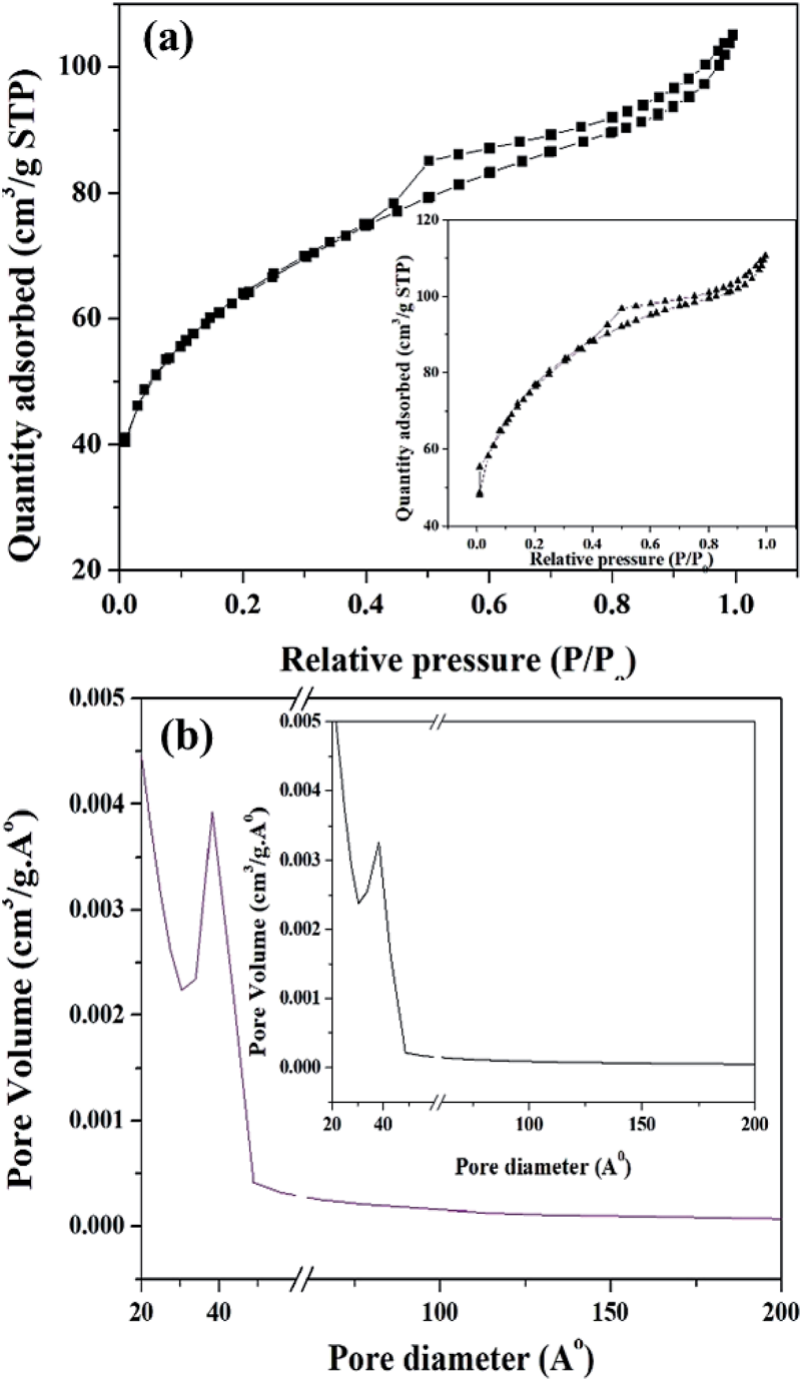
(a) N_2_ adsorption–desorption isotherm and (b) pore size distribution curve of PW_11_Cu/ZrO_2_ and (insets) pure ZrO_2_.

The BET surface area of ZrO_2_ and PW_11_Cu/ZrO_2_ are shown in Table 1. It is clearly seen that the surface area of PW_11_Cu/ZrO_2_ (218 m^2^ g^−1^) is greater than that of zirconia (170 m^2^ g^−1^), because of the supporting, which is as expected. An average pore diameter of 38.25 Å was obtained from the pore size distribution curve (Fig. 9).

**Table 1:** BET surface area of pure ZrO_2_ and PW_11_Cu/ZrO_2_

Material	Surface area (m^2^ g^−1^)
ZrO_2_	170
PW_11_Cu/ZrO_2_	218

### Catalytic activity – oxidation of styrene

**Scheme 1:**
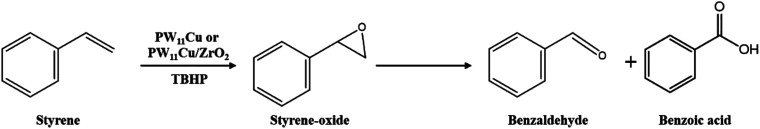
Oxidation of styrene.

A detailed catalytic study was carried out for the oxidation of styrene using the both, unsupported PW_11_Cu as well as PW_11_Cu/ZrO_2_ for a comparative study on the role of support (Scheme 1). The reaction in the absence of the catalyst gives negligible conversion, indicating the necessity of the catalyst for oxidation reaction. Initially, a preliminary reaction was carried out with H_2_O_2_ as the oxidant, and the conversion obtained was insignificant. Hence, further reactions were carried out with TBHP as the oxidant. Reaction parameters like % loading, reaction time, catalyst amount, amount of TBHP and reaction temperature were optimized to give maximum conversion and selectivity of desired product. Each experiment was carried out thrice and the results obtained were reproducible with an error of ±2%. The major products formed were benzaldehyde and styrene-oxide while small amounts of acetophenone and benzoic acid were formed. Small quantities of *tert*-butyl alcohol was formed as by-product as expected.

#### (i) Using PW_11_Cu

**Fig. 10:**
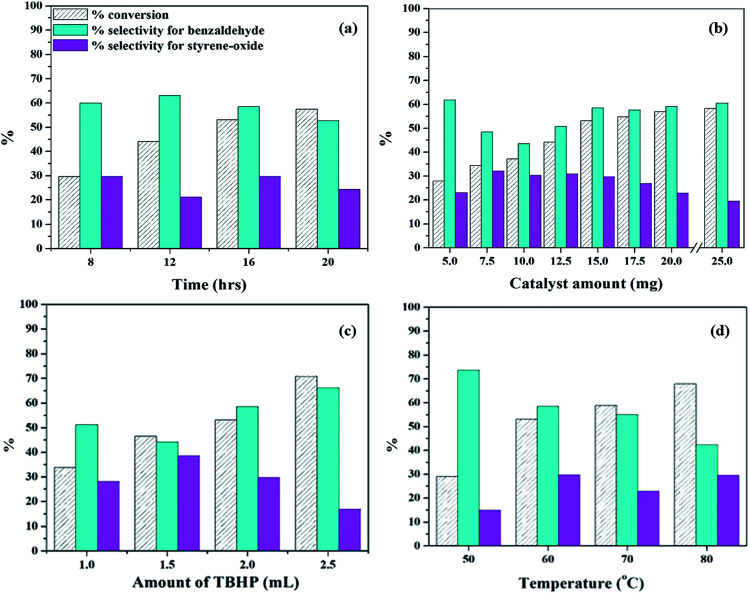
Optimization of parameters for oxidation of styrene (a) effect of time (catalyst amount – 15 mg; temp – 60 °C; TBHP – 2 mL); (b) effect of catalyst amount (time – 16 h; temp – 60 °C; TBHP – 2 mL); (c) effect of amount of TBHP (catalyst amount – 15 mg; temp – 60 °C; time – 16 h); (d) effect of temperature (catalyst amount – 15 mg; time – 16 h; TBHP – 2 mL).

In order to study the effect of time, the reaction was carried out at different times, and the results are presented in Fig. 10a. With increase in time from 8 to 16 h, there is a steady increase in % conversion. At the same time, the selectivity of benzaldehyde decreases and the selectivity for styrene-oxide increases. But when the reaction proceeds further to 20 h, the selectivity of both benzaldehyde as well as styrene oxide, decreases. It is well known that styrene-oxide is the intermediate that is formed during the oxidation of styrene, and the decrease in selectivity of the products may be attributed to complete oxidation of styrene to benzaldehyde and further to benzoic acid. Hence, reaction time was optimized at 16 h.

The reaction was then carried out at different catalyst amounts and the results are presented in table 10b. It can be seen that with increase in catalyst amount from 5 mg to 15 mg, there is a steady increase in conversion of styrene. However, the selectivity of benzaldehyde initially decreases up to 10 mg and then increases, while the selectivity of styrene oxide initially increases and then decreases. This may be because with increase in catalyst amount, the reaction tends to move forward towards completion. Beyond 15 mg, the increase in conversion as well as selectivity of benzaldehyde is negligible. Hence, 15 mg was optimized as the catalyst amount.

The amount of TBHP was then varied to study the effect of oxidant and the results are presented in Fig. 10c. With increase in TBHP amount from 1 mL to 2.5 mL, there is an increase conversion and selectivity of benzaldehyde along with decrease in selectivity for styrene-oxide, which is the expected trend. Excess of TBHP would lead to increase in oxygen concentration, and this would lead to further oxidation of benzaldehyde to benzoic acid, as seen in case of 2.5 mL TBHP. Hence, 2 mL TBHP was considered optimum for the reaction.

Finally, the reaction was carried at different temperatures and the results are presented in Fig. 10d. When the temperature was increased from 50 °C to 60 °C, there is an increase in conversion as well as selectivity of styrene-oxide. But on increasing temperature beyond 60 °C, there is a significant decrease in the selectivity of benzaldehyde as well as styrene-oxide. This may be due to the degradation of TBHP at higher temperatures, thereby resulting in polymerization of styrene. Hence, temperature was optimized at 60 °C.

#### (ii) Using PW_11_Cu/ZrO_2_

**Fig. 11:**
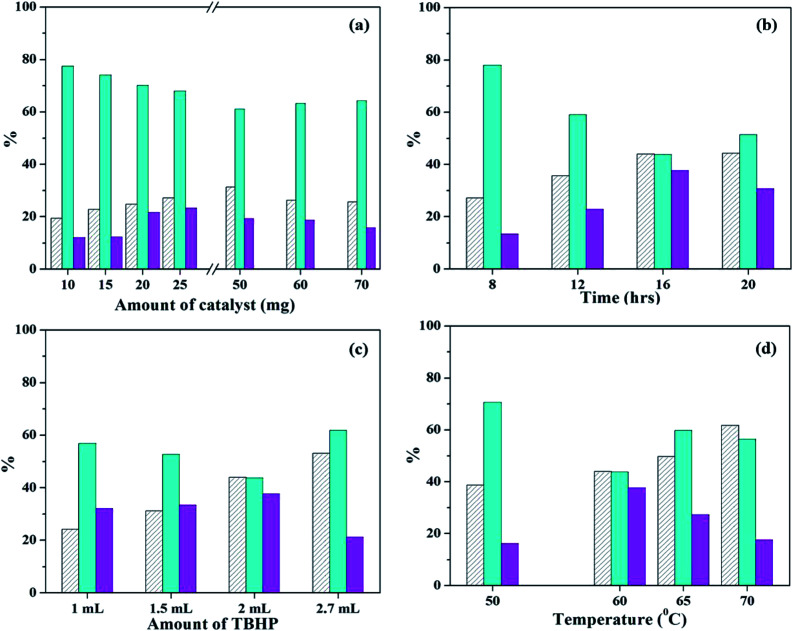
Optimization of parameters for oxidation of styrene (a) effect of catalyst amount (time – 8 h; temp – 60 °C; TBHP – 2 mL); (b) effect of time (catalyst amount – 25 mg; temp – 60 °C; TBHP – 2 mL); (c) effect of amount of TBHP (catalyst amount – 25 mg; temp – 60 °C; time – 16 h); (d) effect of temperature (catalyst amount – 25 mg; time – 16 h; TBHP – 2 mL).

To study the effect of amount of catalyst, the reaction was carried out at varied catalyst amounts keeping other parameters constant (Fig. 11a). The results show that from 15 mg to 25 mg, conversion of styrene, as well as selectivity of styrene oxide increases, however, selectivity of benzaldehyde decreases. On increasing the catalyst amount to 50 mg, there is slight increase in conversion, but selectivity of styrene oxide decreases and that of benzaldehyde increases. Further increase in catalyst amount results in substantial decrease in conversion as well as selectivity of styrene-oxide, which is as expected and may be attributed to blocking of catalytic sites. Hence, 25 mg of the catalyst was considered optimum, which consisted of 6.25 mg of active PW_11_Cu.

Next, reaction time was optimized keeping all other parameters constant (Fig. 11b). As the time is increased from 8 h to 16 h, steady increase in conversion is observed, along with selectivity of styrene-oxide. Further increase in reaction time shows no significant change in either conversion or selectivity of the required product. Therefore, reaction time was optimized at 16 h.

In order to optimized amount of oxidant, the reaction was carried out by adding different quantities of TBHP (Fig. 11c). With increase in TBHP amount from 1 mL to 2 mL, there is a steady increase in conversionof styrene, with increase in styrene-oxide selectivity and decrease in that of benzaldehyde. On further increase in TBHP amount to 2.7 mL, conversion increases, but selectivity of the epoxide decreases, with an increase in selectivity of benzaldehyde. Hence, 2 mL TBHP was considered optimum for the reaction.

Finally, the reaction temperature was optimized by carrying out the reaction at different temperatures (Fig. 11d). On increasing the temperature from 50 °C to 60 °C, there is increase in conversion and styrene oxide selectivity. But further increase in temperature results in significant decrease of selectivity of both epoxide as well as aldehyde, with formation of unwanted by-products in the form of polymers. This is due to decomposition of TBHP at higher temperatures. Therefore, reaction temperature was optimized at 60 °C.

The reaction conditions for oxidation of styrene using both catalysts were optimized as follows: (I) for PW_11_Cu: catalyst amount – 15 mg; reaction time – 16 h; TBHP – 2 mL; reaction temperature – 60 °C; (II) for PW_11_Cu/ZrO_2_: catalyst amount – 25 mg (active species – 6.25 mg); reaction time – 16 h; TBHP – 2 mL; reaction temperature – 60 °C. It is necessary to bring to notice that in the present systems, optimization has been carried out keeping in priority the selectivity of styrene-oxide. These conditions may be varied by the chemist depending on the requirement of the products.

### Test for leaching and heterogeneity

Stability of the catalyst during the course of the reaction is of utmost importance as leaching of active species would make the catalyst unappealing. Hence, PW_11_Cu was tested for leaching of copper. Absence of characteristic absorption peaks for Cu(ii) in the UV-Vis spectrum of reaction mixture confirmed that both catalysts do not leach during the course of the reaction. In case of PW_11_Cu/ZrO_2_, absence of blue colour on treating the reaction mixture with ascorbic acid clearly indicated that the catalyst remains intact during the course of the reaction.

To check the heterogeneity of PW_11_Cu, the reaction was first run for 12 h, after which the catalyst was filtered out from the reaction mixture. The reaction was then allowed to proceed further up to 16 h. Similar set of experiments were carried out for PW_11_Cu/ZrO_2_. No change in the conversion as well as selectivity of the products (Table 2) indicates that the both catalysts are truly heterogeneous in nature.

**Table 2:** Test for heterogeneity

Catalyst	% conversion	% selectivity	
		Benzaldehyde	Styrene-oxide
aPW_11_Cu (12 h)	44	63	21
aFiltrate (16 h)	45	64	22
bPW_11_Cu/ZrO_2_ (12 h)	40	44	21
bFiltrate (16 h)	40	46	20

a Catalyst amount: 15 mg; temp: 60 °C; TBHP: 2 mL.

b Catalyst amount: 25 mg; temp: 60 °C; TBHP: 2 mL.

### Effect of support

A comparison of the activity of unsupported and supported catalysts under optimized conditions as well as the active amount of PW_11_Cu present in PW_11_Cu/ZrO_2_ is shown in Table 3. It can be seen that supported catalyst gives more than double the conversion along with much better selectivity of epoxide, as compared to unsupported one under optimized conditions, which can be explained as follows: (i) homogeneous dispersion of the PW_11_Cu over ZrO_2_, due to which more active sites are available for catalysis, and (ii) the overall acidity of the catalyst.

**Table 3:** Effect of support

Catalyst	% conversion	% selectivity		Turn over number (TON)
		Benzaldehyde	Styrene-oxide	
aPW_11_Cu	44	63	21	1102
bPW_11_Cu	21	67	20	1102
cPW_11_Cu/ZrO_2_	44	44	37	2309

a Catalyst amount – 15 mg; TBHP – 2 mL; temperature – 60 °C; time – 16 h.

b Catalyst amount –6.25 mg; TBHP – 2 mL; temperature – 60 °C; time – 16 h.

c Catalyst amount – 25 mg (active amount of PW_11_Cu-6.25 mg); TBHP – 2 mL; temperature – 60 °C; time – 16 h.

It is observed that the conversion for both, supported as well as unsupported catalyst under optimized conditions is the same. However, it is interesting to note that in case of PW_11_Cu/ZrO_2_, the active species present is less than half the amount to that of PW_11_Cu alone. This is the superiority of the supported catalyst.

The total number of acidic sites of the support, PW_11_Cu and PW_11_Cu/ZrO_2_ were calculated by potentiometry and the results are presented in Table 4. There is a phenomenal increase in the overall acidic strength in case of supported catalyst compared to the individual materials, and hence the higher catalytic activity. This is further confirmed by a detailed kinetic study of the reaction using PW_11_Cu as well as PW_11_Cu/ZrO_2_ as catalysts.

**Table 4:** Acidic sites determined by potentiometry

Material	Acidic strength (mV)	Types of acidic sites			Total no. of acidic sites
		Very strong	Strong	Weak	
ZrO_2_	53	0	0.5	0.8	1.3
PW_11_Cu	20	0	0.1	0.3	0.4
PW_11_Cu/ZrO_2_	140	0.1	0.1	1.0	1.2

### Kinetic studies

**Fig. 12:**
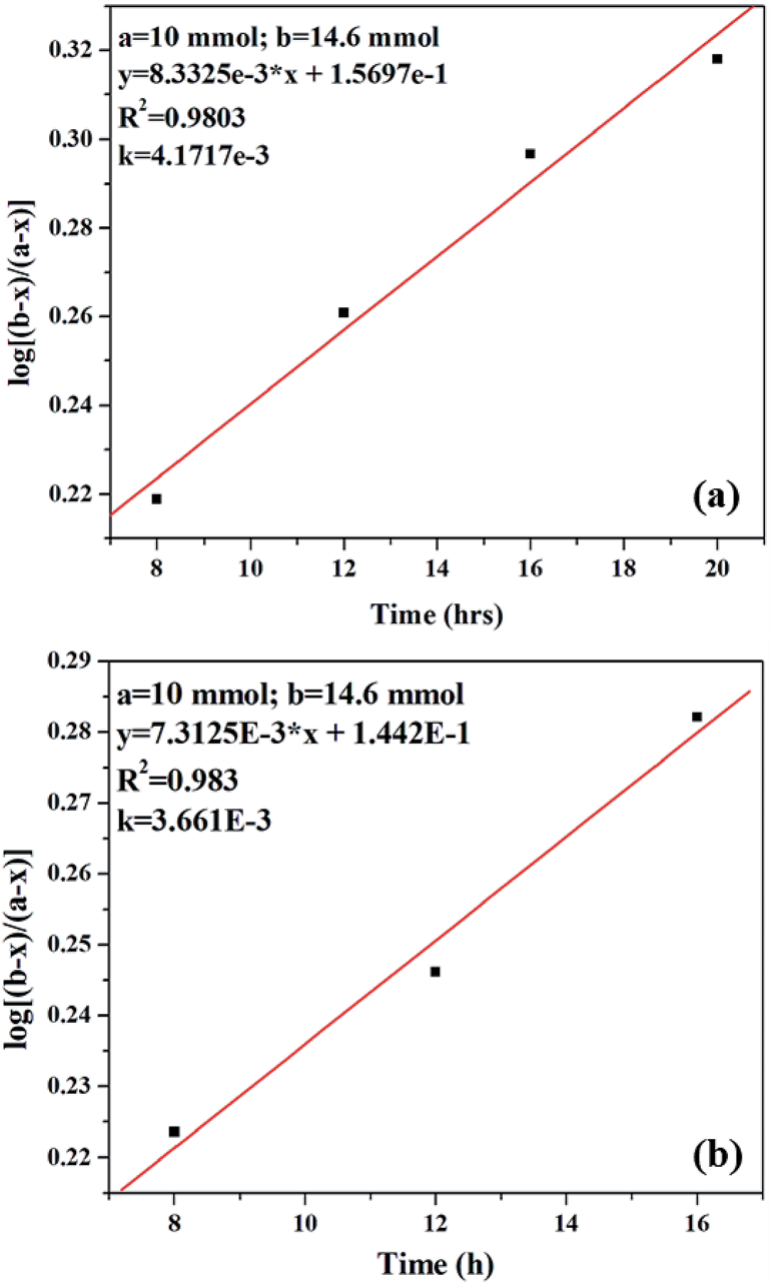
Plot of log[(*b* − *x*)/(*a* − *x*)] *versus* time for (a) PW_11_Cu and (b) PW_11_Cu/ZrO_2_.

Kinetic study of the reaction was carried out in detail for both the catalysts in order to comprehend the effect of each component on the rate of reaction. Experiments were carried out with different initial concentrations of styrene and TBHP, keeping the catalyst amount constant. Eqn (1) establishes a relation between the individual concentration of the reactants and time.1
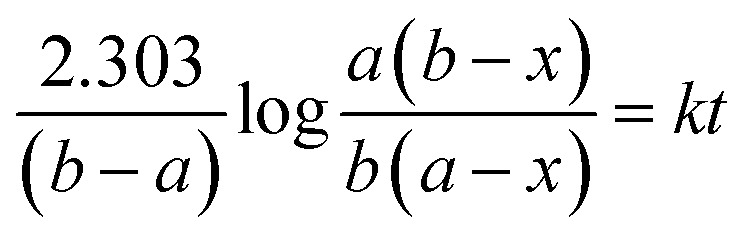
where ‘*a*’ is the initial concentration of styrene, ‘*b*’ is the initial concentration of TBHP and ‘*x*’ is the concentration at time *t*. A plot of log[(*b* − *x*)/(*a* − *x*)] *versus* time shows a straight line for both catalysts (Fig. 12), indicating that in both the cases, the reaction is first order with respect to styrene and TBHP, individually.^50,51^

**Fig. 13:**
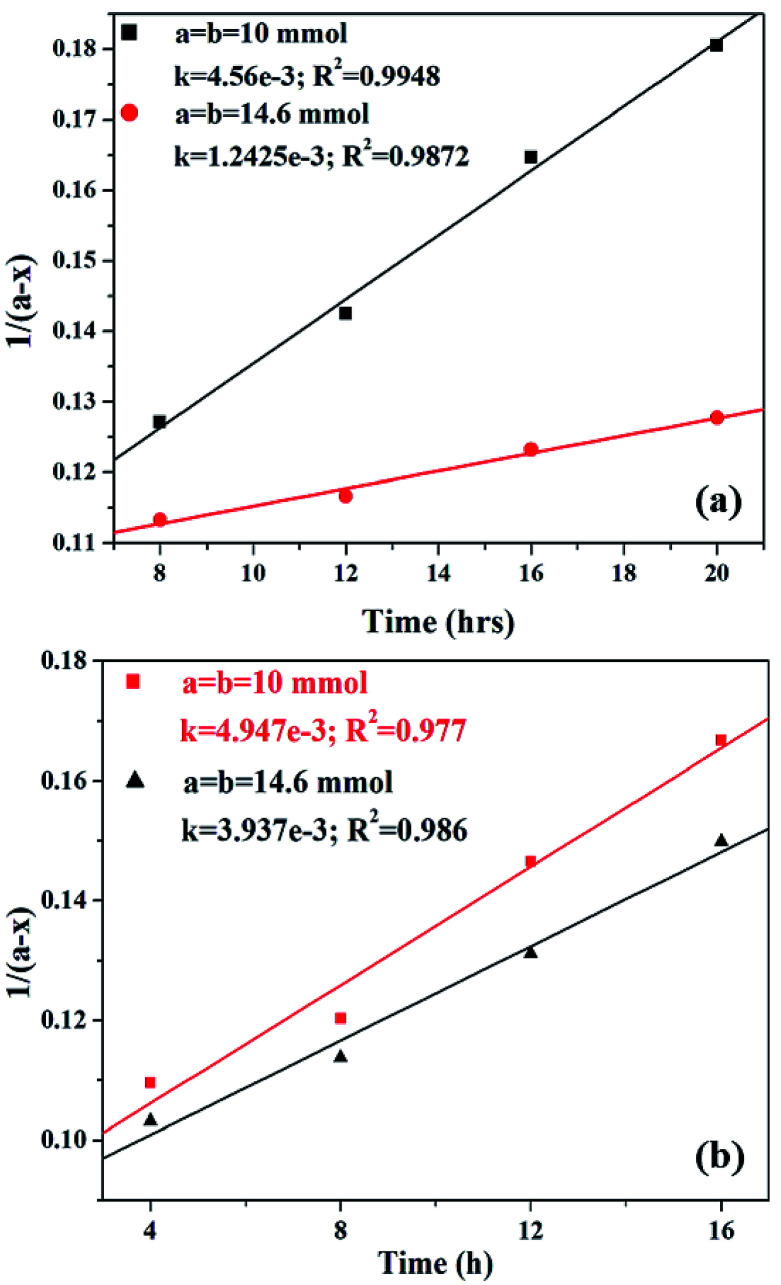
Plot of 1/(*a* − *x*) *versus* time for (a) PW_11_Cu and (b) PW_11_Cu/ZrO_2_.

Alternatively, an experiment was then carried out keeping the concentration of styrene as well as TBHP the same. Eqn (2) establishes a relation between the concentration and time.2
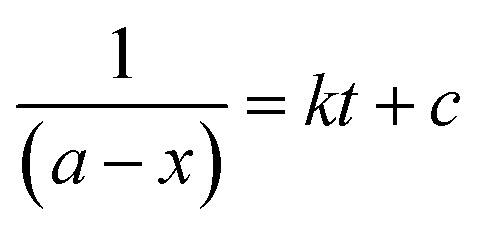
where ‘*a*’ is the initial concentrations of styrene and TBHP and ‘*x*’ is the conversion of styrene at time *t*. As in the previous case, a plot of 1/(*a* − *x*) *versus* time shows a linear relationship for both the systems, (Fig. 13) indicating that the reaction follows second order overall with respect to concentration of styrene and TBHP in both the cases.^50,51^

**Fig. 14:**
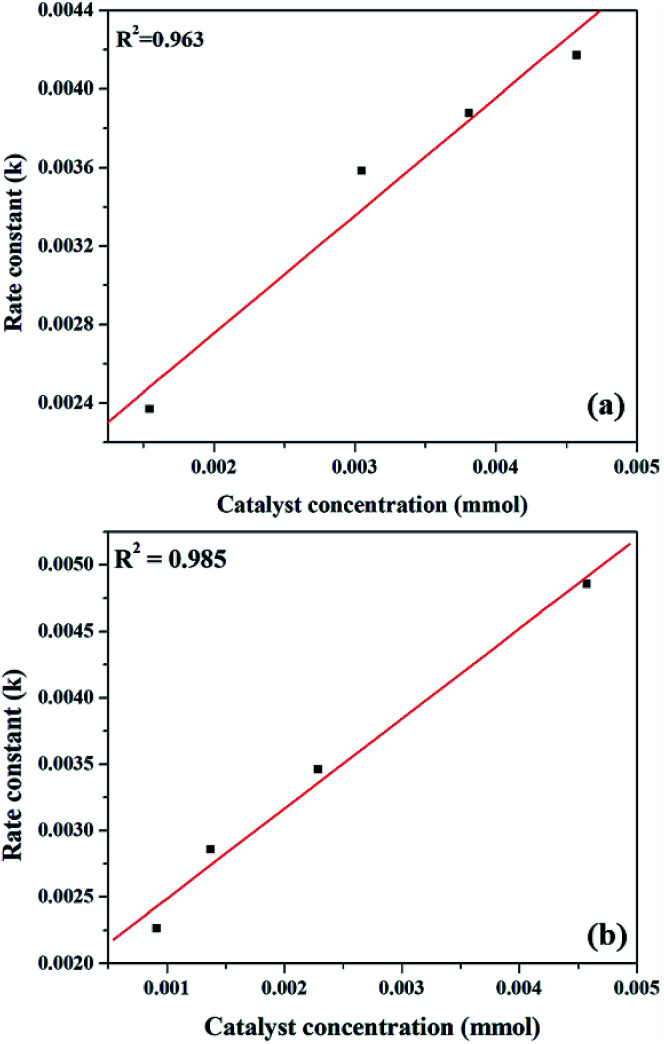
Plot of rate of reaction *versus* catalyst concentration for (a) PW_11_Cu and (b) PW_11_Cu/ZrO_2_.

The effect of reaction rate with respect to catalyst concentration was studied wherein rate constants were plotted at different catalyst concentrations and shown in Fig. 14. When the concentration of PW_11_Cu is increased from 1.545 × 10^−3^ mmol to 4.573 × 10^−3^ mmol, there is a linear increase in rate of the reaction. Similarly, increase in concentration of active species in PW_11_Cu/ZrO_2_ from 9.15 × 10^−4^ mmol to 4.573 × 10^−3^ mmol, also shows linearity. This indicates that the reaction follows first order kinetics with respect to catalyst concentration as well.^50,51^

### Determination of activation energy

**Fig. 15:**
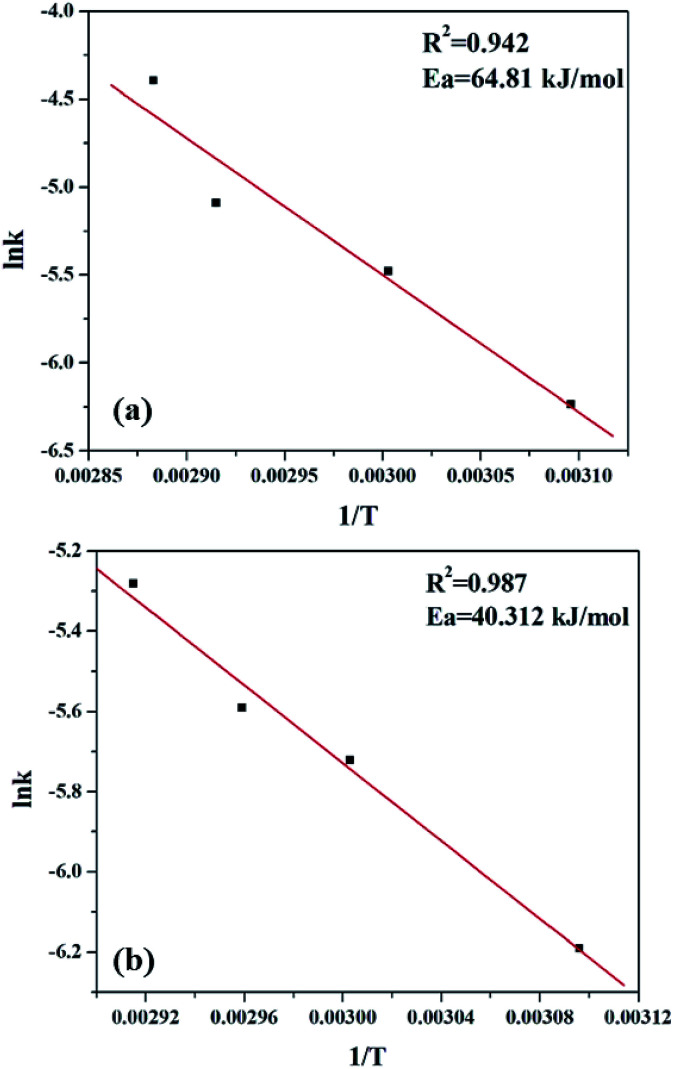
Plot for determination of activation energy for (a) PW_11_Cu and (b) PW_11_Cu/ZrO_2_.

As most oxidation reactions are highly sensitive to temperatures, the effect of temperature plays a very significant role. When temperature is increased from 323 K to 353 K, a gradual increase in the conversion of styrene is seen. Thus, 1/*T* shows a linear relationship with lnk (Fig. 15), and the activation energy was evaluated using the Arrhenius equation.

3 *k* = *A* e^−*E*_a_/*RT*^

As seen clearly, the present system is a two phase system, and hence it is necessary to clarify whether the reaction is truly governed by a chemical step or it is merely limited to mass transfer or diffusion. Usually, mass transfer limited reactions show *E*_a_ in the range of 10–15 kJ mol^−1^, as opposed to a true chemical reaction which shows *E*_a_ > 25 kJ mol^−1^.^50,52^ Significantly higher activation energy of 64.81 kJ mol^−1^ for PW_11_Cu and 40.31 kJ mol^−1^ in case of PW_11_Cu/ZrO_2_ indicates that the reaction is truly governed by a chemical step and also that both the catalysts have been exploited to its maximum capacity. Interestingly, lower activation energy of PW_11_Cu/ZrO_2_ compared to PW_11_Cu also showcases the superiority of the supported system over unsupported one. Thus, the conclusions obtained from kinetic studies fittingly support that of catalysis that PW_11_Cu/ZrO_2_ is a better catalytic system than PW_11_Cu.

2,6-Di-*tert*-butyl-4-methyl phenol was used as the radical scavenger to gain an idea on the mechanism and know the intermediate in the reaction for both the systems. The radical scavenger was added 12 h after the reaction was started and then the reaction was allowed to proceed to completion, and the results are present in Table 5. No significant change in the conversion and selectivity in both the cases, indicates that the intermediate formed is a radical. The slight increase in conversion and selectivity of benzaldehyde may be due to presence of the radical intermediate after immediate addition of scavenger, as in the present case, the oxidant used is TBHP, which is a well-known radical initiator. This confirms that the reaction proceeds *via* radical mechanism, which is as expected.^53^

**Table 5:** Inhibition experiment with 2,6-di-*tert*-butyl-4-methylphenol as radical scavenger

Condition	% conversion	% selectivity	
		Benzaldehyde	Styrene-oxide
After 12 h	44^a^/40^b^	63/44^b^	21/21^b^
After 16 h (after scavenger addition)	46^a^/41^b^	65^a^/44^b^	20^a^/22^b^

a PW_11_Cu: catalyst amount: 15 mg; temp: 60 °C; TBHP: 2 mL.

b PW_11_Cu/ZrO_2_: catalyst amount: 25 mg; temp: 60 °C; TBHP: 2 mL.

### Recycle study

**Fig. 16:**
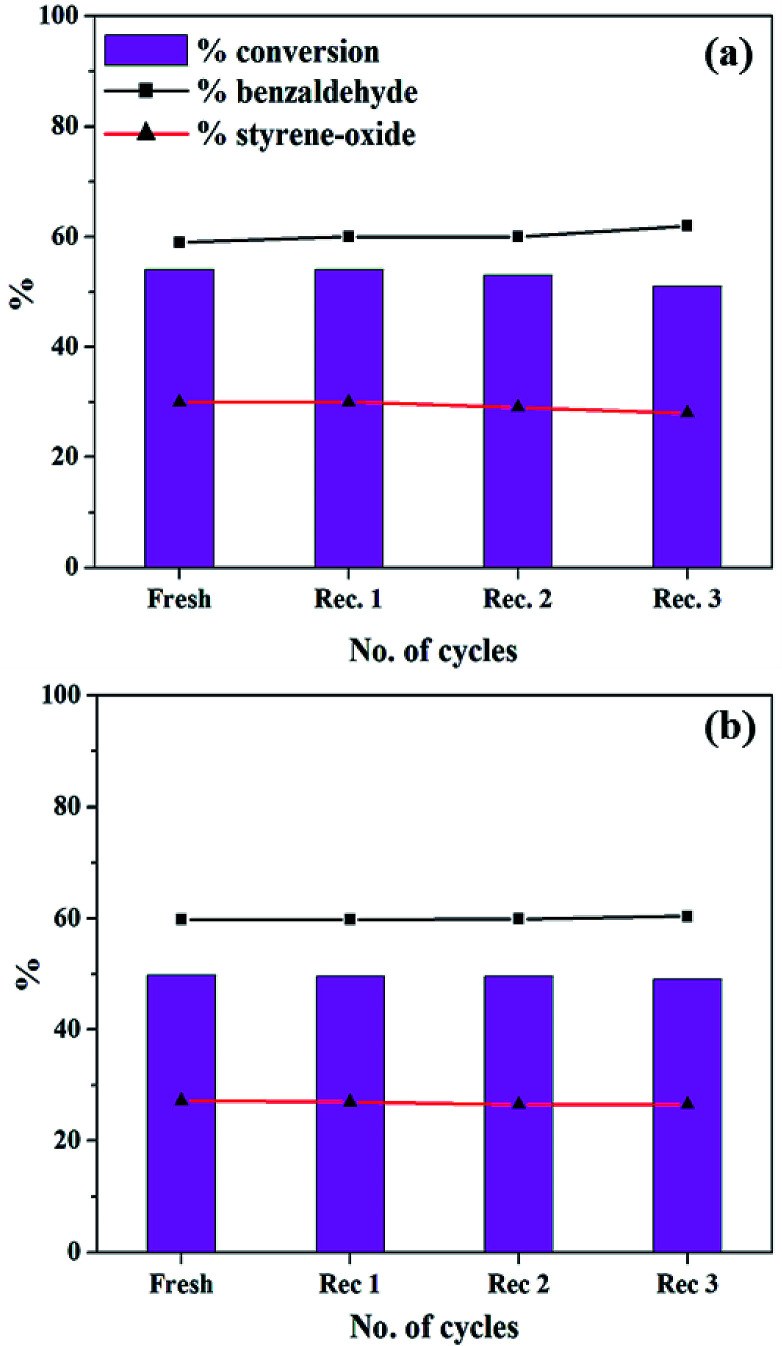
Recycle study for (a) PW_11_Cu (catalyst amount: 15 mg; temp: 60 °C; TBHP: 2 mL) and (b) PW_11_Cu/ZrO_2_ (catalyst amount: 25 mg; temp: 60 °C; TBHP: 2 mL).

In order the regenerate the catalysts, the reaction mixture was centrifuged to separate the catalyst, and this was followed by washing with methanol and drying. The regenerated catalyst was used in the same reaction under optimized conditions and the results are presented in Fig. 16.

In both the cases, same conversion and selectivity for the products are obtained even after multiple cycles indicating that the catalyst remains stable during the course of the reaction and that it can be reused for multiple times. A slight decrease in conversion in the third cycle may be due to loss of the catalyst during the recovery.

### Characterization of regenerated catalyst

**Fig. 17:**
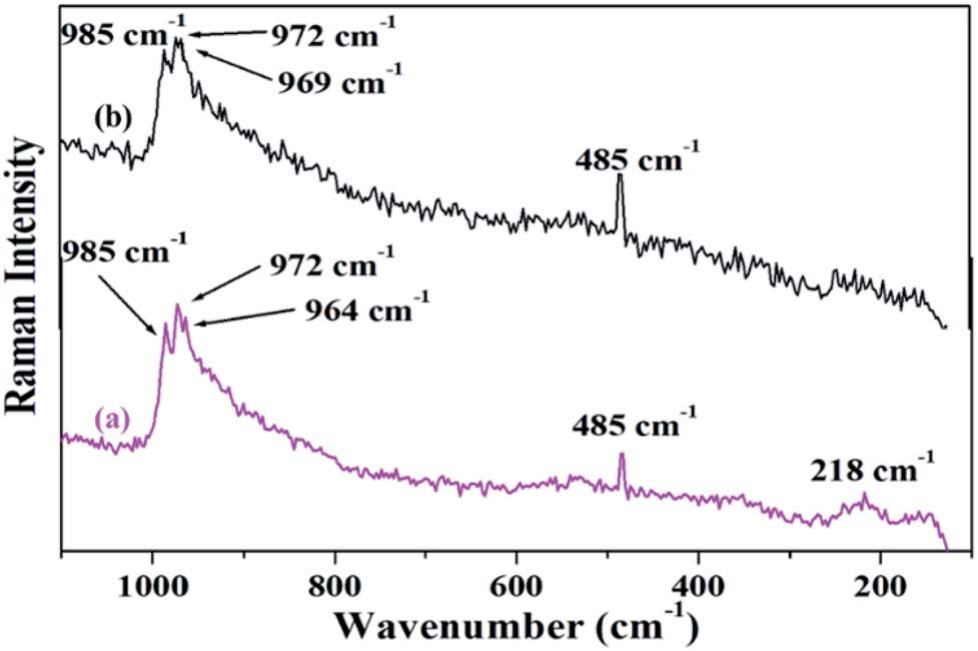
ESR spectra of (a) PW_11_Cu and (b) Rec. PW_11_Cu.

Regenerated PW_11_Cu was characterized by ESR and FT-IR spectroscopy while regenerated PW_11_Cu/ZrO_2_ was characterized by FT-IR, FT-Raman, and powder XRD. Fig. 17 shows the ESR spectra of fresh and recycled PW_11_Cu. It can be clearly seen that both the spectra are similar, as the five line spectrum is obtained back in the recycled PW_11_Cu confirming that the environment around Cu remains intact after regeneration.

The FT-IR spectrum of Rec. PW_11_Cu (Table 6) shows characteristic bands at 1103, 1056, 964, 887 and 813 cm^−1^ corresponding to P–O, W=O, and W–O–W stretching frequencies respectively. The Cu–O band can be clearly seen at 483 cm^−1^. No significant shifting of bands clearly indicates that the catalyst does not degrade and the structural morphology of the complex remains unchanged during the course of the reaction. Similarly, Rec. PW_11_Cu/ZrO_2_ shows bands at 1101, 1057, 960 cm^−1^ corresponding to P–O and W=O frequencies, and 3417, 1665 and 1413 cm^−1^ respectively for O–H, H–O–H and O–H–O frequencies of ZrO_2_. The slight decrease in the sharpness of the bands in recycled catalyst may be due to impurities that remain on the catalyst after recycling.

**Table 6:** FT-IR frequencies of fresh and recycled catalysts

Catalyst	FT-IR frequencies (cm^−1^)						
	P–O	W=O	W–O–W	Cu–O	O–H	H–O–H	O–H–O
PW_11_Cu	1103	964	887	489	—	—	—
	1061		810				
Rec. PW_11_Cu	1103	964	887	483	—	—	—
	1056		813				
PW_11_Cu/ZrO_2_	1103	952	813	—	3417	1631	1402
	1064						
Rec. PW_11_Cu/ZrO_2_	1101	960	—	—	3417	1665	1413
	1057						

**Fig. 18:**
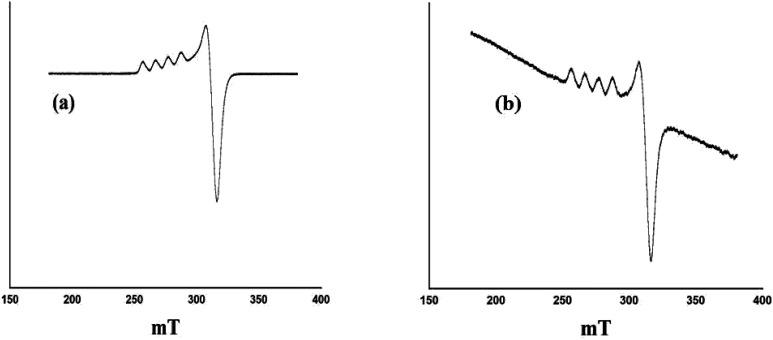
FT-Raman frequencies of (a) fresh and (b) recycled PW_11_Cu/ZrO_2_.

The FT-Raman spectra of fresh and recycled PW_11_Cu/ZrO_2_ (Fig. 18) shows that all the characteristic peaks of the fresh catalyst are retained in the recycled one. Thus, FT-IR and FT-Raman spectra indicate that the structure remained intact even after regeneration.

**Fig. 19:**
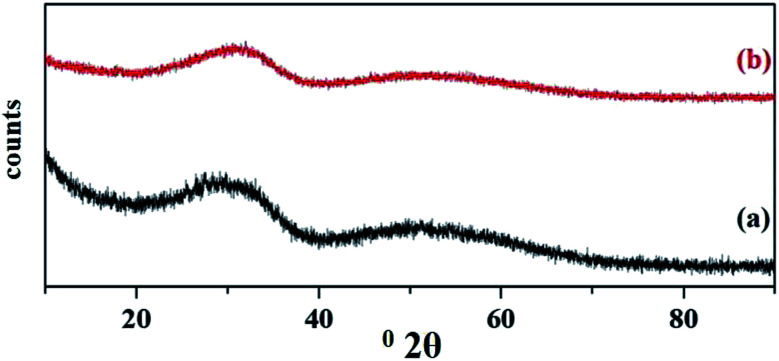
Wide angle powder XRD of (a) fresh and (b) regenerated PW_11_Cu/ZrO_2_.

The wide angle powder XRD patterns of fresh and recycled PW_11_Cu/ZrO_2_ are shown in Fig. 19. No appreciable change in the powder XRD of regenerated catalyst compared to the fresh one indicates that the structure remains unaffected after the regeneration of catalyst.

## Conclusions

Mono-copper substituted phosphotungstate supported on hydrous zirconia was successfully synthesized by wet impregnation technique and characterized using various techniques. FT-IR and FT-Raman confirmed the intact structure of PW_11_Cu as well as support even after impregnation, while powder XRD confirmed homogeneous dispersion. TPR indicated strong chemical interactions between PW_11_Cu and the support, and ^31^P MAS NMR confirmed the same. The supported catalyst showed better activity as compared to the unsupported one towards oxidation of styrene with TBHP oxidant, where higher conversion (PW_11_Cu/ZrO_2_: 44%, PW_11_Cu: 21%) and higher selectivity towards styrene-oxide (PW_11_Cu/ZrO_2_: 37%, PW_11_Cu: 20%) along with substantially higher TON (PW_11_Cu/ZrO_2_: 2309, PW_11_Cu: 1102) showcased the superiority of supported catalyst. Both the catalysts were recycled and reused up to three cycles with negligible loss in catalytic activity. Detailed kinetic studies showed first order dependence with respect to both substrates individually, and an overall second order dependence. Substantially lower activation energy of PW_11_Cu/ZrO_2_ (40.31 kJ mol^−1^) compared to PW_11_Cu (64.81 kJ mol^−1^) further affirmed the superiority of supported catalyst.

## Conflicts of interest

There are no conflicts to declare.

## Supplementary Material
